# Cytokine Storm Syndrome Responsive to IL-1 Inhibition in Trisomy 21

**DOI:** 10.1155/2024/9946401

**Published:** 2024-03-28

**Authors:** Aimee Magnarelli, Julia Shalen, Maria J. Gutierrez

**Affiliations:** Division of Pediatric Allergy, Immunology and Rheumatology, Johns Hopkins University School of Medicine, Baltimore, MD, USA

## Abstract

**Background:**

Cytokine storm syndromes (CSS) are life-threatening systemic inflammatory disorders caused by immune system dysregulation. They can lead to organ failure and are triggered by various factors, including infections, malignancy, inborn errors of immunity, and autoimmune conditions. Trisomy 21 (TS21), also known as Down syndrome, is a genetic disorder associated with immune dysfunction, increased infection susceptibility, and inflammation. While TS21 has been linked to infectious-triggered hyperinflammation, its role as a primary cause of CSS has not been confirmed. *Case Presentation*. We present a case of a 16-year-old male with TS21 with fever, rash, joint pain, and abdominal symptoms. Extensive investigations ruled out infections, autoimmune conditions, malignancies, and inborn errors of immunity as triggers for a CSS. The patient's symptoms improved with treatment using IL-1 inhibition and corticosteroids.

**Conclusions:**

This case reinforces that TS21 is an immune dysregulation disorder and highlights the importance of considering CSS in TS21 patients, even when triggers are unclear. The positive response to IL-1 inhibition in this patient suggests that dysregulation of the IL-1 superfamily and the NLRP3 inflammasome may contribute to CSS in TS21. This finding raises the possibility of using IL-1 inhibition as a treatment approach for CSS in TS21 patients.

## 1. Background

Cytokine storm syndromes (CSS) are a group of life-threatening systemic hyperinflammation disorders caused by abnormal immune activation or dysregulation. Patients present with fever, fatigue, anorexia, diarrhea, skin rashes, arthralgias, myalgias, and neuropsychiatric changes [1]. Elevated inflammatory markers, ferritin, and d-dimer levels, increased serum cytokine levels (e.g., IFN-g, IL-6, IL-10, and soluble CD25), hypertriglyceridemia, blood-count abnormalities (e.g., leukocytosis or leukopenia, anemia, and thrombocytopenia) are common, hemophagocytic macrophages can be observed in the bone marrow contributing to cytopenias [2–4], and excessive cytokine production can lead to multiorgan failure and mortality.

There are multiple known triggers of CSS, including infections, malignancy, and inborn errors of immunity (IEI), producing a CSS named hemophagocytic lymphohistiocytosis (HLH) [5]. A clinically similar hemophagocytic CSS in autoinflammatory and autoimmune disorders is called macrophage activation syndrome (MAS) [6–8]. Iatrogenic CSS, including cytokine release syndromes after immunotherapy (e.g., OKT3 infusions), and chimeric antigen receptor (CAR) T-cell treatment are additional examples [9–11]. During the COVID-19 pandemic, SARS-CoV-2 infections have also been linked to CSS, with alteration in innate immunity and excess cytokine production, including IL-2, IL-6, IL-7, IL-10, INF-*γ*, and TNF-*α* [12]. Inflammatory mechanisms mediating CSS vary and include dysregulation of innate, humoral, or cellular immune pathways [1, 9, 10]. Determining the cause is important as treatments vary greatly based on predisposing conditions and possible pathways and mechanisms involved.

Trisomy 21 (TS21) is a genetic condition associated with increased infection susceptibility, inflammation, autoimmunity, and hematologic malignancies. TS21 has been related to infectious-triggered hypercytokinemia (e.g., after SARS-CoV-2 and influenza infections) [13–15]. Here, we present a case of TS21 presenting with a CSS without identifiable triggers, successfully treated with IL-1 inhibition, further proving that TS21 may be associated with life-threatening systemic hyperinflammation.

## 2. Case Presentation

A 16-year-old Caucasian male with a history of TS21, hypothyroidism, and alopecia presented to our hospital with fever of unknown origin for 13 days, associated with arthralgias, rash, and abdominal pain with emesis. His initial evaluation in the emergency room showed increased ferritin (15,095 ng/mL), c-reactive protein (CRP) (12.1 mg/dL), erythrocyte sedimentation rate (ESR) (22 mm/h), and AST (63 U/L) with thrombocytopenia (146 K/cu mm), intermittent hypothermia, and hypoxia. Additional labs revealed a significant hyperinflammatory state with persistently elevated serum inflammatory markers CRP (12.3 mg/dL) and ferritin (25,754 ng/mL), markedly elevated interleukin-18 (IL-18) (343,991 pg/mL), soluble CD25 (sIL-2) (26,450 pg/mL), and pro-brain natriuretic peptide (pBNP) (1,355 pg/mL) serum levels but inappropriately decreasing ESR (9 mm/hr) and fibrinogen (164 mg/dL), concerning for a CSS. He had no travel history outside of the United States. During his hospitalization, he was evaluated for possible infectious, malignant, autoimmune, autoinflammatory, and immunodeficiency underlying causes. Despite a comprehensive infectious workup, including bacterial blood cultures, SARS-CoV-2 and HIV serologies, and screening for other viruses (cytomegalovirus, Epstein–Barr virus, adenovirus, and human herpesvirus 6), tick-borne illnesses (*Rickettsia rickettsii,* Ehrlichiosis), and other infections (*Rickettsia typhi*), no infectious cause was found. The oncologic evaluation included a bone marrow biopsy that was unrevealing. Hair trichoscopy for Gray Hair syndromes, including Chediak–Higashi, Griscelli, and Hermansky–Pudlak, which can be associated with primary immunodeficiencies, was normal. He had a history of organ-specific autoimmunity (alopecia areata and hypothyroidism), but no rheumatological disorders associated with MAS were identified. Specifically, there was no history of arthritis or other signs of autoimmune or autoinflammatory disease. His autoantibody profile (ANAs, ANCAs, ASO, anti-Sm, anti-dsDNA, and antiphospholipid antibodies) was negative, and he had normal complement (C3 and C4) levels. A chromium release assay to evaluate NK function was normal, immunoglobulin levels and antibody responses (tetanus, diphtheria, and pneumococcal IgG) were adequate, and whole-exome sequencing (WES) was negative for variants in genes knowingly associated with HLH or IEI. His hematologic workup showed acute anemia, thrombocytopenia, and lymphopenia with decreased CD4+ T cells and B cells. However, lymphocyte counts were normal before his current illness.

Upon hospital admission, he was acutely treated with oral and intravenous vitamin K, cryoprecipitate for fibrinogen <100, and pulse steroids (1 gram for three days). In addition, he received intravenous (IV) anakinra, starting with an initial dose of 100 mg twice a day, which then increased to 200 mg twice a day before being gradually tapered off over one week.

**Figure 1:**
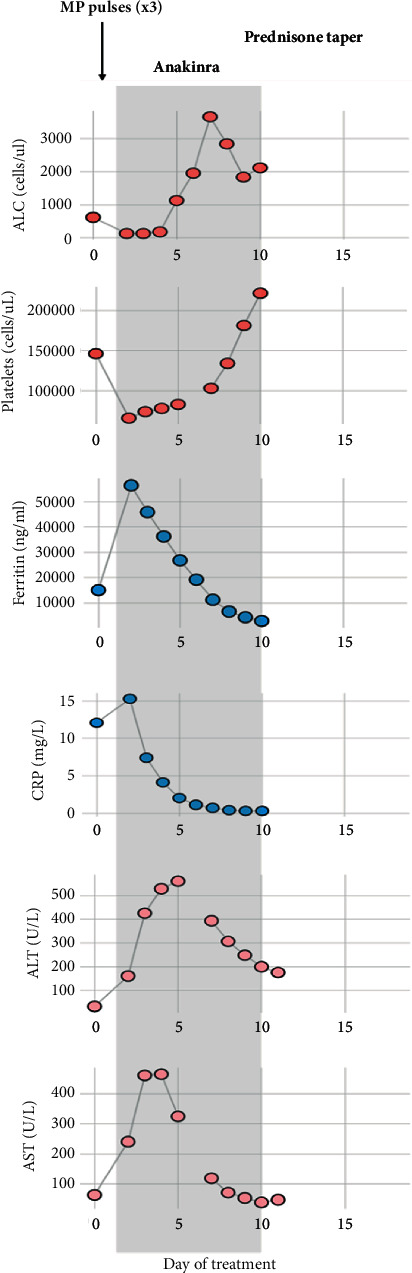
Inflammatory response during anti-IL-1 therapy. Daily monitoring of blood cell counts (absolute lymphocyte counts (ALC) and platelets), serum c-reactive protein (CRP), ferritin, and liver transaminases (AST and ALT) showed rapid improvement after therapy with methylprednisolone (MP) and intravenous anakinra (in grey shade) was initiated. Anemia and marked elevations of B-type natriuretic (BNP) and soluble IL-2 (sIL-2) also reverted with therapy (data not shown).

Notably, during the treatment with IV anakinra, the patient's fevers improved, his liver enzymes and inflammatory markers started to decrease, and there were clinical signs of improvement (Figure 1). After his initial treatment with three pulses of methylprednisolone (1 gr IV on three consecutive days), concurrently with IV anakinra, he was transitioned to a prednisolone taper. After treatment discontinuation, his symptoms have not relapsed, and labs show no evidence of inflammation.

## 3. Discussion

Trisomy 21 is a multisystem, complex genetic disease associated with multiple types of immune dysfunction. For instance, genes encoding four of the six interferon receptor subunits (IFNAR1, IFNAR2, IFNGR2, and IL-10 receptor 2) are located in chromosome 21, leading to increased responsiveness to interferon signaling. This results in the upregulation of proinflammatory cytokines and chemokines downstream interferon pathways. Upregulation of interferon signaling is also associated with increased numbers of natural killers, cytotoxic CD8+ T cells, and decreased numbers of B cells. In addition, lymphocyte abnormalities (e.g., reduced T-cell receptor excision circle counts, reduced lymphocytes, and defects in B-cell differentiation leading to a decrease in switch memory B cells), impaired chemotaxis, and abnormal regulatory T-cell (Treg) generation and function are also described in TS21 patients [14–17]. Clinically, individuals with TS21 have increased susceptibility to infections [18, 19] and are prone to autoimmune complications. For instance, dysregulated activity of Toll-like receptors and interferon has recently been considered to play a role in the development of autoimmunity in TS21 [20]. Additionally, their risk of hematologic malignancies is elevated, being 10- to 20-fold higher than in the euploid population [21].

Here, we present the case of a 16-year-old male with TS21 presenting with life-threatening CSS. Notably, despite extensive investigation for potential triggers, including infections, previously described systemic autoimmune and autoinflammatory conditions, malignancy, and inborn errors of immunity, no additional underlying causes of CSS were identified throughout the patient's illness. It is plausible that the symptoms were triggered by an undetected infection or were a postinfectious complication in the context of TS21. Similar cases of CSS have been reported in TS21 patients following SARS-CoV-2 infections, potentially linked to increased IFN activity [15, 16]. In our case, it is possible that the patient had a COVID-19 infection that went undetected, as seen in some children presenting with MIS-C [22]. Nevertheless, an expanded infectious disease workup did not reveal any infection, suggesting that TS21 might have been the primary cause of the patient's acute hyperinflammation.

Notably, the patient exhibited a significant elevation of IL-18, and the symptoms successfully responded to treatment with anakinra and corticosteroids. While systemic corticosteroids are known to modulate multiple immunological pathways effectively, the patient's clinical features and response to IL-1 inhibition suggest an inflammasome-mediated inflammatory disease. This is particularly relevant, given that elevations in inflammasome-related cytokines in the IL-1 superfamily (e.g., IL-1, IL-18, and IL-36) and the overactivation of inflammasomes are common phenomena in other forms of CSS [23, 24]. Indeed, anakinra, which targets IL-1, has become a key drug used as a first-line treatment of patients with HLH and other CSS such as rheumatological disease-associated MAS [25]. Interestingly, increased IL-1*β* expression has also been described in TS21 patients [13, 26], and although the data are limited, increased NLRP3 activity has been proposed as a mechanism of immune dysregulation in TS21 [17]. This suggests that further research is needed to fully understand the role of inflammasome-mediated immune dysregulation in TS21 and the potential benefit of IL-1 inhibitors in the treatment of CSS in this patient population.

## 4. Conclusion

This case highlights that TS21 is an immune dysregulation disorder that may present with life-threatening CSS, prompts questions about whether dysregulation of IL-1 and inflammasome pathways contribute to the pathogenesis of CSS in TS21, and raises the possibility of utilizing IL-1 inhibition with anakinra as a treatment approach for the treatment of CSS in TS21 patients.

## Data Availability

The data supporting the current study are available from the corresponding author upon request.

## Abbreviations

CSS: Cytokine storm syndromes
HLH: Hemophagocytic lymphohistiocytosis
MAS: Macrophage activation syndrome
CAR: Chimeric antigen receptor
TS21: Trisomy 21
CRP: c-reactive protein
ESR: Erythrocyte sedimentation rate
MIS-C: Multisystem inflammatory syndrome in children
pBNP: Pro-brain natriuretic peptide
WES: Whole-exome sequencing
Treg: Regulatory T cell.
